# Three-Dimensional Thermal Conductive Modeling of Hybrid Thermal Conductive Powders Filled POK-Based Composites

**DOI:** 10.1021/acsomega.6c00448

**Published:** 2026-06-18

**Authors:** Lutfiye Altay, Mehmet Sarikanat, Mehmet Mete Tokgoz, Yoldas Seki

**Affiliations:** 1 Department of Mechanical Engineering, 37509 Ege University, İzmir 35100, Turkey; 2 İzmir Eğitim Sağlık Sanayi Yatırım A.Ş., Turgutlu, Manisa 45420, Turkey; 3 Department of Chemistry, Dokuz Eylul University, İzmir 35390, Turkey

## Abstract

This study investigates the development of polyketone (POK)–based composites reinforced with various thermally conductive fillers—including synthetic graphite (SG), carbon fiber (CF), carbon nanotubes (CNTs), and hexagonal boron nitride (BN)—and combinations thereof. Single-, binary-, and ternary-filler systems were fabricated to examine the influence of filler type, morphology, concentration, and interactions on the resulting thermal conductivity. A three-dimensional analytical thermal conduction model was developed to predict the effective thermal conductivity of the composites, incorporating filler anisotropy, fiber orientation, interfiller interactions, and multifiller synergy. Fiber orientation distributions were obtained using Moldex3D simulations and integrated into the model. The in-plane thermal conductivity increased from 0.21 W/mK for neat POK to as high as 9.16 W/mK with 40 wt % synthetic graphite, while carbon fiber–reinforced composites reached 8.54 and 1.12 W/mK in in-plane and through-plane directions, respectively. BN–CNT hybrid systems exhibited synergistic improvements even at low loadings. Model predictions showed strong agreement with experimental values, validating the reliability of the proposed approach. The graphical comparison demonstrates strong agreement between model predictions and experimental results across all filler systems, with deviations remaining within ± 10% for fiber-reinforced composites and within ± 5% for particle-filled systems. These findings offer practical guidance for the design and optimization of lightweight, thermally conductive composites for advanced thermal management applications.

## Graphical abstract

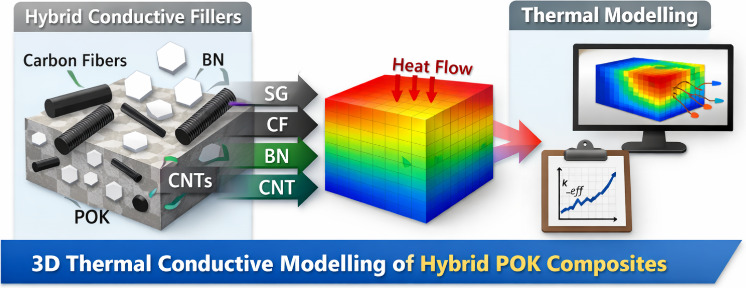

## 1 Introduction

Polymers possess intrinsically low thermal conductivity—typically between 0.2 and 0.3 W/m·K—which significantly limits their applicability in devices and systems that require efficient heat dissipation. The rapid progress of microelectronics, portable technology, automotive power electronics, and aerospace systems has accelerated the need for materials capable of managing localized heat generation while maintaining low weight, dimensional stability, and mechanical robustness.^1–3^ As electronic components continue to scale down and power density increases, the risk of thermal hotspots grows, making effective thermal management a critical design challenge.

Conventional thermal management solutions rely heavily on metals such as aluminum and copper due to their high thermal conductivity. However, their relatively high density limits their use in weight-sensitive applications, including mobile devices, unmanned systems, electric vehicles, and aircraft electronics. As a result, thermally conductive polymer composites have attracted considerable attention as lightweight alternatives offering tunable thermal and mechanical properties, chemical resistance, processability, and compatibility with mass-production techniques such as injection molding.

To narrow the performance gap between highly conductive metals and polymers, researchers have developed polymer composites incorporating thermally conductive fillers such as metals and carbon-based materials.^3–6^ A broad range of high-performance reinforcements—including graphene, carbon fiber, boron nitride, carbon nanotubes, and graphite—can be integrated into thermoplastic matrices to achieve substantially enhanced heat transfer capabilities.^7–11^ The distribution, orientation, and dispersion quality of these fillers within the polymer matrix are critical determinants of the resulting thermal and mechanical properties. Owing to the anisotropic nature of many conductive fillers, thermal conductivity in these composites is often significantly higher in the in-plane direction than in the through-plane direction.^3,10,12,13^ This inherent anisotropy must be carefully addressed during composite design, especially for applications that require efficient heat dissipation in multiple directions.

Several theoretical models, including Hashin–Shtrikman bounds, the Maxwell–Eucken equation, percolation models, and numerical techniques such as finite element analysis (FEA) have been developed to predict the effective thermal conductivity of polymer composites incorporating fillers.^14^ Studies on fiber-reinforced thermoplastic composites produced by injection molding show that fiber orientation strongly influences influence on thermal conduction. However, additional factors—such as fiber aspect ratio, interfacial adhesion between the fiber and matrix, filler connectivity, packing behavior, and composite porosity—must also be incorporated to improve the accuracy and reliability of thermal conductivity predictions.^15–22^ Despite growing research interest, the anisotropic thermal conductivity of polymer composites reinforced with multiple filler types remains insufficiently investigated. A critical factor governing thermal transport is the formation of conductive networks, in which synergistic interactions arising from differences in filler size, shape, and composition ratio can substantially enhance overall thermal conductivity.^3^

A previous study conducted by Seki et al.^3^ experimentally investigated thermally conductive POK composites containing CNT, BN, and graphite fillers, primarily focusing on thermal conductivity enhancement and material performance. Building on that experimental foundation, the present study extends the work by developing a comprehensive three-dimensional predictive thermal-conductivity framework for hybrid-filler-reinforced POK composites. The proposed approach integrates (i) flow-dependent fiber orientation distributions obtained from Moldex3D simulations, (ii) anisotropic thermal properties of fillers and reinforcements, (iii) filler morphology and shape-factor effects, and (iv) hybrid multifiller interaction parameters within a unified analytical model. Unlike previous studies that mainly focused on experimental characterization or simplified orientation assumptions, this work combines process simulation and analytical thermal modeling to evaluate the influence of injection molding-induced orientation on anisotropic thermal conductivity behavior in multifiller POK composites

## 2 Materials and Method

### 2.1 Materials

Polyketone (POK) M330, supplied by HYOSUNG Chemical Corporation, was used as the matrix polymer. Recycled carbon fiber (CF) (CF.OS.A) was obtained from Apply Carbon. Synthetic graphite (SG), TIMREX KS 44, was purchased from IMERYS Graphite & Carbon (Switzerland). Boron nitride (BN) powder (CFX 1020) was supplied by Momentive. Multiwalled carbon nanotubes (CNTs) were sourced from Nanografi (Türkiye) with the following specifications: 92% purity and an outer diameter of 8–10 nm.

### 2.2 Fabrication of POK based composite granules

Composite granules were fabricated using a corotating, intermeshing twin-screw extruder with an L/D ratio of 48 (Leistritz 27 MAXX). After exiting the extruder die and passing through a water bath, the polymer strands were pelletized using a strand pelletizer. CF, SG, BN, CNT, and a BN–CNT blend were incorporated into the POK matrix at weight fractions of 10–40 wt %, 10–40 wt %, 1–3 wt %, 1–3 wt %, and 1.5/1.5 wt %, respectively. The composites were compounded using a corotating twin-screw extruder operated at a screw speed of 500 rpm, with a throughput of approximately 28 kg/h. The temperature profile along the extrusion zones was maintained primarily between 290–300 °C, while the die temperature was set to 280 °C. The feeding zone temperature was adjusted to 40 °C. The POK matrix was fed through the main feeder, while the thermally conductive fillers were introduced into the extruder using side feeders in order to achieve more stable feeding and improved filler dispersion during melt compounding. The extruded polymer strands and the morphology of the resulting composite granules are shown in Figure 1. After extrusion, the strands were water-cooled and pelletized prior to injection molding.. Test specimens for thermal conductivity measurements were produced from the composite granules using an injection molding machine (Bole BL90EK).

**Figure 1:**
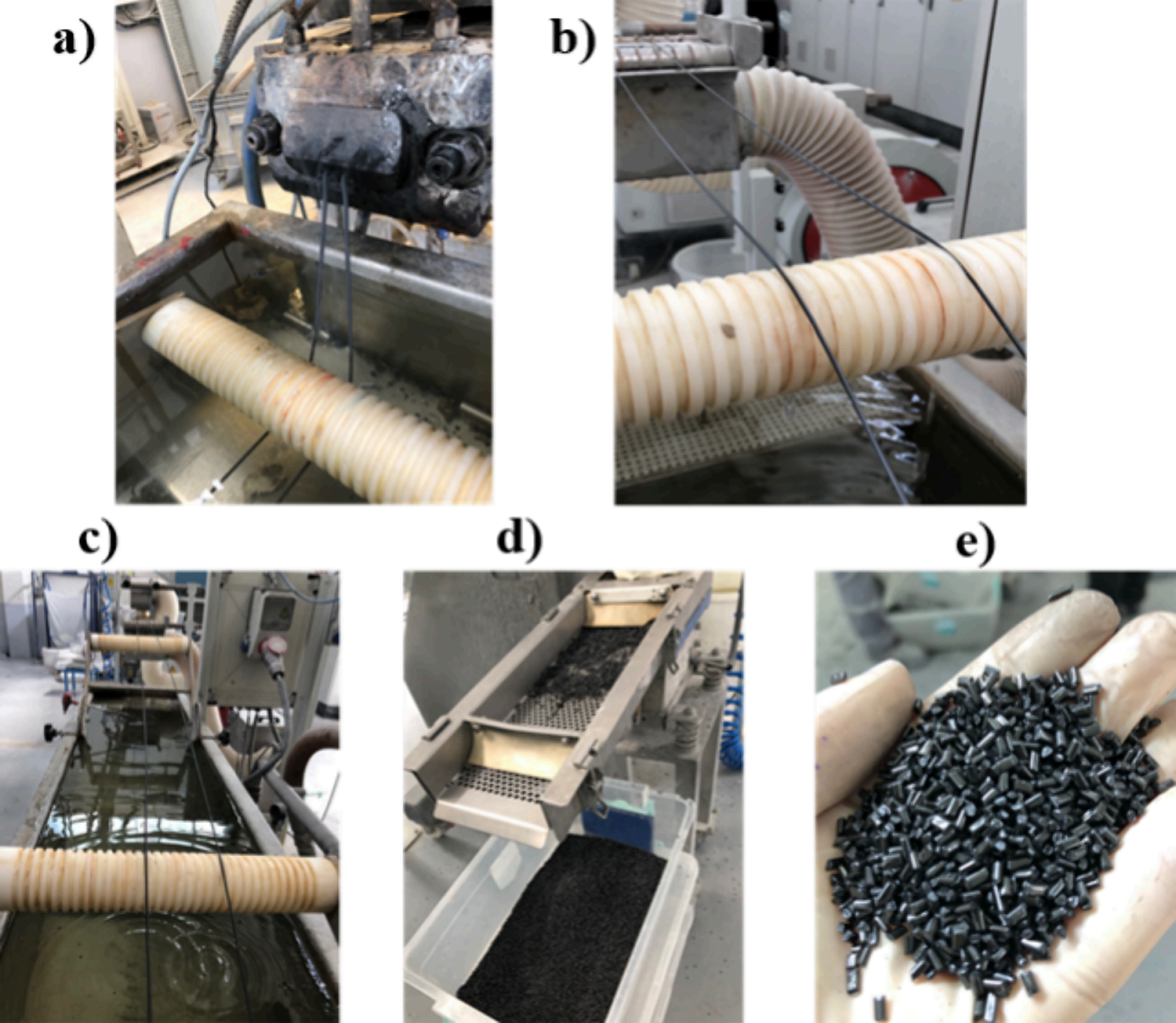
Fabrication process of POK-based composites: (a) Extrusion of polymer strands from the twin-screw extruder die, (b) cooling of the extruded strands in a water bath, (c) pelletizing process using a strand pelletizer, (d) collection of the composite granules, and (e) macro-morphology of the final POK-based composite granules ready for injection molding.

### 2.3.Thermal Conductivity Measurements

In-plane and through-plane thermal conductivities were determined from thermal diffusivity measurements using a Xenon Flash Instrument (DXF 200) in accordance with ASTM E1461–07.

### 2.4 Carbon Fiber Orientation Characterization

Three-dimensional fiber orientation distributions were predicted using Moldex3D R14.0, which solves the generalized Navier–Stokes equations coupled with fiber orientation evolution based on the Folgar–Tucker model. Four different gate locations were analyzed to assess their influence on fiber alignment.^23^ The simulations were conducted under conditions identical to those used during experimental injection molding in order to ensure direct comparability between numerical predictions and measured thermal conductivities.

#### 2.4.1 Numerical Setup, Boundary Conditions, and Mesh Independence

The injection molding simulations employed a three-dimensional tetrahedral mesh. A mesh convergence study was performed by progressively refining the mesh until variations in predicted fiber orientation tensors and temperature fields were less than 2% between successive refinements. The final mesh consisted of approximately 1.2 million elements, which provided an optimal balance between numerical accuracy and computational efficiency.

The simulations were performed by prescribing a constant melt inflow rate of *Q*_*in*_ = 20 cm^3^/s, corresponding to an inlet velocity of approximately *u*_*in*_ = 0.05 m/s. A zero-gauge-pressure condition (*p* = 0) was applied at the vent/outlet, while no-slip boundary conditions (u = 0) were imposed on all mold walls. Here, P denotes the static pressure and u represents the fluid velocity vector.

The melt inlet temperature was set to 240 °C, whereas the mold wall temperature was fixed at 80 °C for unfilled and particle-filled systems and 120 °C for CF-containing systems. Heat transfer at the mold wall was modeled using a convective boundary condition with a heat transfer coefficient of *h* = 5.0 × 10^3^ W/m^2^K.

The initial conditions were defined as *T*_0_ *=*240 °C, *p*_0_ = 0, u_0_ = 0, and isotropic fiber orientation A_0_ = I/3, where I denotes the second-order identity tensor. The time step was set to 10^–4^ s during the filling stage and 10^–3^ s during the packing/cooling stage.

Four different gate locations were evaluated to assess their influence on fiber alignment and the resulting anisotropic thermal conductivity. The gate configuration used in the experimental specimens corresponded to Flow Direction 3, as determined by comparing numerical predictions with experimental data.

#### 2.4.2 Integration of Fiber Orientation into the Thermal Conductivity Model

Fiber orientation tensors exported from Moldex3D were incorporated into the analytical thermal conductivity model using a laminate analogy approach, treating the composite as a stack of laminae with locally varying fiber orientations defined by angles (θ, Φ). Each finite element was assigned a local orientation tensor describing the statistical alignment of fibers within that region.

The effective thermal conductivity of each lamina was calculated based on the local orientation distribution, and the overall conductivity of the composite was obtained by thickness-wise averaging. This approach allows the model to capture spatial variations in fiber alignment induced by flow conditions, particularly near the gate and along the melt flow direction.

A mathematical model was developed to predict how filler and reinforcement orientation influence thermal conductivity. This model allows for the straightforward calculation of the thermal conductivity of multifiller and/or reinforcement composites. To this end, the following model was formulated.

$$K_{j}=k_{p}\frac{1+\overset{n}{\underset{f=1}{\sum }}A_{f}\times B_{f,j}{Ø}_{f}+\overset{m}{\underset{r=1}{\sum }}A_{r}\times B_{r,j}\times {Ø}_{r}}{1+\overset{n}{\underset{f=1}{\sum }}B_{f,j}\times \psi_{f}\times {Ø}_{f}-\overset{m}{\underset{r=1}{\sum }}B_{r,j}\times \psi_{r}\times {Ø}_{r}}\left(j=1,2\right)$$

The parameters *B*_*r,j*_,ψ_*f*_ and ψ_*r*_ quantify directional contributions of fillers and reinforcements. In this model,*B*_*r,j*_, ψ_*f*_ and ψ_*r*_ were calculated using the following formulas;

$$B_{r,j}=\frac{\frac{k_{r,j}}{k_{p}}-1}{\frac{k_{r,j}}{k_{p}}-A_{r}}\left(j=1,2\right)$$

$$\psi_{f}\overset{∼}{=}1+\frac{1-{Ø}_{mf}}{{Ø}_{mf}^{2}}\times {Ø}_{f}\psi_{f}\overset{∼}{=}1+\frac{1-{Ø}_{mr}}{{Ø}_{mr}^{2}}\times {Ø}_{r}$$

where *K*_1_ and *K*_2_ represent the thermal conductivity values of the composite material in the in-plane and through-plane directions, respectively. Ø*x* parameters denote volume fractions: Ø_*f*_ for filler and Ø*r* for reinforcement. Additionally, Ø_*mf*_ and Ø_*mr*_ indicate the maximum filler and reinforcement ratios, respectively. *k*_*p*_ represents the thermal conductivities of the polymer matrix. *k*_f,1_ and *k*_f,2_ represent the thermal conductivities of the fillers in the planar and thickness directions, respectively. *k*_r,1_ and *k*_r,2_ denote the thermal conductivities of the reinforcements in the planar and thickness directions, respectively. The subscripts ‘f’ and ‘r’ represent filler and reinforcement, respectively. *A*_*f*_ and *A*_*r*_ denote the shape factors for the filler and reinforcement, respectively. In the model calculations, the shape factors were set to 1.20 for BN, 1.41 for SG, and 3.12 for CF. The Ø_*mf*_ values were taken as 0.506 for BN, 0.614 for SG, while the value for carbon fiber (Ø_*mr*_ *)* was set to 0.441.

#### 2.4.3 Determination of *k*_*r*,1_ Value from Fiber Orientation Data

Fiber orientation tensors were exported from Moldex3D. A laminate analogy approach was applied, treating local regions as stacked laminae with unique fiber orientations defined by angles (θ, Φ). The finite element model used for the simulation comprised tetrahedral elements,where each element was associated with an orientation tensor describing the fiber alignment at that specific position. For the calculation of the subsample’s thermal conductivity, only the elements contained within the subsample region were considered.

The laminate analogy approach was used to evaluate the thermal conductivity of short fiber-reinforced composites. In this approach, the composite is modeled as a stack of laminae with varying fiber orientations and length. The fiber orientation is defined by a pair of angles (θ, Φ), as shown in Figure 2.

**Figure 2:**
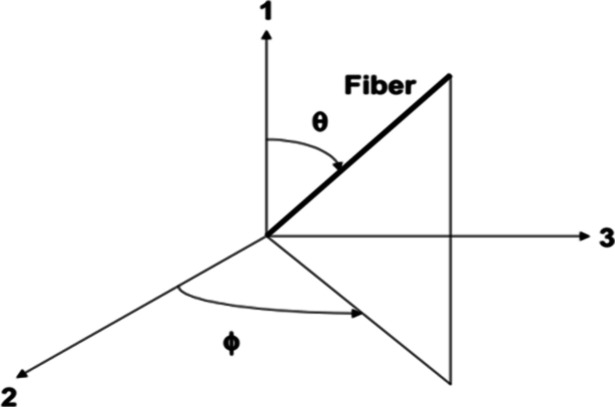
Fiber angles.

The fiber length and orientation distribution density functions, *f­(L)* and *g­(*θ*)*, are

$$f\left(L\right)={abL}^{b-1}\exp\left(-{aL}^{b}\right)forL>0$$

$$g(\theta )=(\sin\theta {)}^{2p-1}(\cos\theta {)}^{2q-1}/\int_{\theta \min}^{\theta \max}(\sin\theta {)}^{2p-1}(\cos\theta {)}^{2q-1}d\theta$$

where a and b are size and shape parameters, respectively, for determining the size and shape of fiber length distribution curves. p and q are the shape parameters which can be used to determine the shape of fiber orientation distribution curve and are in the range of 0 ≤ θ_min_ ≤ θ ≤ π/2.

*K*_1_ represents the thermal conductivity in the direction parallel to the fiber orientation, while *K*_2_ represents the thermal conductivity in the direction perpendicular to the fiber orientation. The corresponding expressions are provided below.

$$K_{1}=\frac{1+2{\alpha \mu }_{1}{Ø}_{r}}{1-\mu_{1}{Ø}_{r}}\times k_{p}$$

$$K_{2}=\frac{1+2{\alpha \mu }_{2}{Ø}_{r}}{1-\mu_{2}{Ø}_{r}}\times k_{p}$$

where α *= L/d*_*f*_ and *d*_*f*_ shows fiber diameter.

μ_1_ and μ_2_ can be obtained from the following equations;

$$\mu_{1}=\frac{K_{r1}/K_{p}-1}{K_{r1}/K_{p}-2\alpha }$$

Here, the following equations were obtained

$$K_{1}^{\prime }=K_{1}\cos^{2}\;\theta +K_{2}sin^{2}\theta$$

$$k_{r,1=\int_{L=L\min}^{L\max}\int_{\theta =\theta \min}^{\theta \max}K_{1}^{\prime }f\left(L\right)g\left(\theta \right)dLd\theta }$$

$$\mu_{2}=\frac{K_{r2}/K_{p}-1}{K_{r2}/K_{p}-2}$$

where *K*_*r1*_ and *K*_*r2*_ represent the thermal conductivities of the fiber parallel to the fiber axis and perpendicular (crosswise) to the fiber axis, respectively.

The method used to calculate thermal conductivity values from flow simulation data is schematically presented in Figure 3.

**Figure 3:**
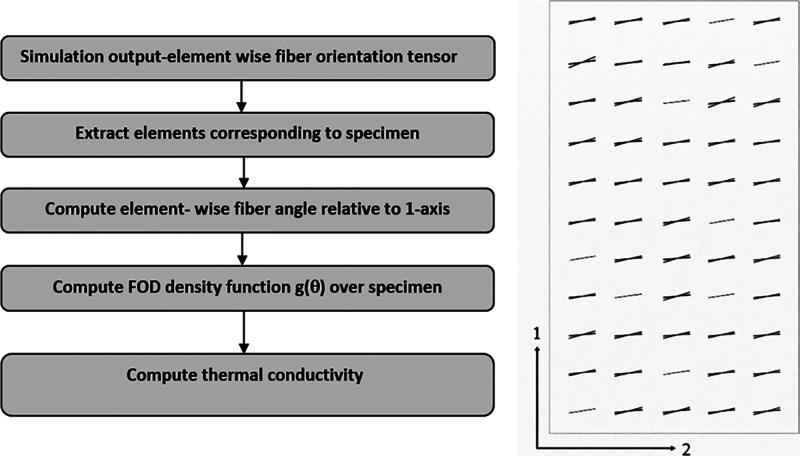
Schematic of thermal conductivity calculation from flow simulation data.

## 3 Results and Discussions

### 3.1 Influence of Filler Type and Loading on Thermal Conductivity

Table 1 summarizes the in-plane and through-plane thermal conductivity values of neat polyketone (POK) and its composites containing various thermally conductive fillers, including synthetic graphite (SG), carbon fiber (CF), carbon nanotubes (CNT), boron nitride (BN), and a hybrid BN–CNT system. Neat POK exhibited a low thermal conductivity of 0.21 W/m·K in both directions. Incorporating SG led to a substantial increase in in-plane conductivity, reaching 9.16 W/m.K at 40 wt % SG, whereas the through-plane conductivity increased only moderately to 0.92 W/m.K. This pronounced discrepancy highlights the strong anisotropic behavior of SG-filled composites, which is likely attributable to the planar alignment of SG particles during melt processing.

**Table 1:** Thermal Conductivity Values of POK Composites (In-Plane and Through-Plane)

**sample**	**in-plane** **(W/m**·**K)**	**through-plane** **(W/m**·**K)**
**POK**	0.21 ± 0.01	0.21 ± 0.01
**POK-10SG**	2.17 ± 0.20	0.35 ± 0.02
**POK-20SG**	3.97 ± 0.32	0.48 ± 0.04
**POK-30SG**	7.02 ± 0.65	0.71 ± 0.06
**POK-40SG**	9.16 ± 0.95	0.92 ± 0.07
**POK-10CF**	1.98 ± 0.02	0.41 ± 0.03
**POK-20CF**	3.54 ± 0.04	0.54 ± 0.05
**POK-30CF**	6.85 ± 0.08	0.87 ± 0.07
**POK-40CF**	8.54 ± 0.07	1.12 ± 0.11
**POK-1CNT**	0.77 ± 0.06	0.37 ± 0.03
**POK-2CNT**	1.05 ± 0.12	0.51 ± 0.04
**POK-3CNT**	1.94 ± 0.02	0.72 ± 0.07
**POK-1BN**	0.45 ± 0.03	0.34 ± 0.02
**POK-2BN**	0.98 ± 0.07	0.41 ± 0.03
**POK-3BN**	1.12 ± 0.10	0.49 ± 0.04
**POK-1.5BN-1.5CNT**	1.57 ± 0.12	0.5 ± 0.04

Similarly, CF-reinforced composites exhibited significant improvements in both in-plane and through-plane thermal conductivity, reaching 8.54 W/m·K and 1.12 W/m·K, respectively, at 40 wt %. This behavior suggests the formation of a more interconnected, three-dimensional filler network that enhances heat transport in multiple directions. CNT-filled composites provided a comparatively balanced improvement, yielding conductivities of 1.94 W/m·K (in-plane) and 0.72 W/m·K (through-plane) at 3 wt %. These results highlight the relatively isotropic dispersion of CNTs and their ability to form efficient conductive pathways even at low loading levels.

BN-based composites exhibited moderate enhancements in thermal conductivity, achieving 1.12 W/m·K in the in-plane direction and 0.49 W/m·K in the through-plane direction at 3 wt %. Although these values are lower than those of carbon-based composites, BN offers the distinct advantage of electrical insulation, making it suitable for applications requiring effective thermal management without electrical conductivity. The hybrid composite containing 1.5 wt % BN and 1.5 wt % CNT demonstrated clear synergistic effects, reaching thermal conductivities of 1.57 W/m·K (in-plane) and 0.50 W/m·K (through-plane). This improvement is likely attributed to the complementary interaction between BN’s plate-like structure and the high-aspect-ratio conductive pathways formed by CNTs.

These findings collectively underscore the importance of filler type, concentration, and morphology in tailoring the thermal conductivity of POK composites. Specifically, SG provided the highest directional (in-plane) enhancement, while CF offered superior through-plane conductivity and a more interconnected heat conduction pathway.

### 3.2 Effect of Injection Molding Parameters on Thermal Conductivity

Fiber orientation is the most critical parameter influencing the thermal conductivity of fiber-reinforced composites, and it is strongly affected by injection molding conditions. Consequently, the careful selection and control of processing parameters are essential for achieving the desired thermal performance. In this study, fiber orientations were predicted using the commercial simulation software Moldex3D R14.0. To determine the optimal gate location, the mold was evaluated under four different gating configurations. The resulting fiber orientation data were then incorporated into the proposed model to calculate the effective thermal conductivity values. Figures 4–6 present the fiber orientation distributions in the x-, y-, and *z*-directions for the POK-20CF composite under each feeding condition.

**Figure 4:**
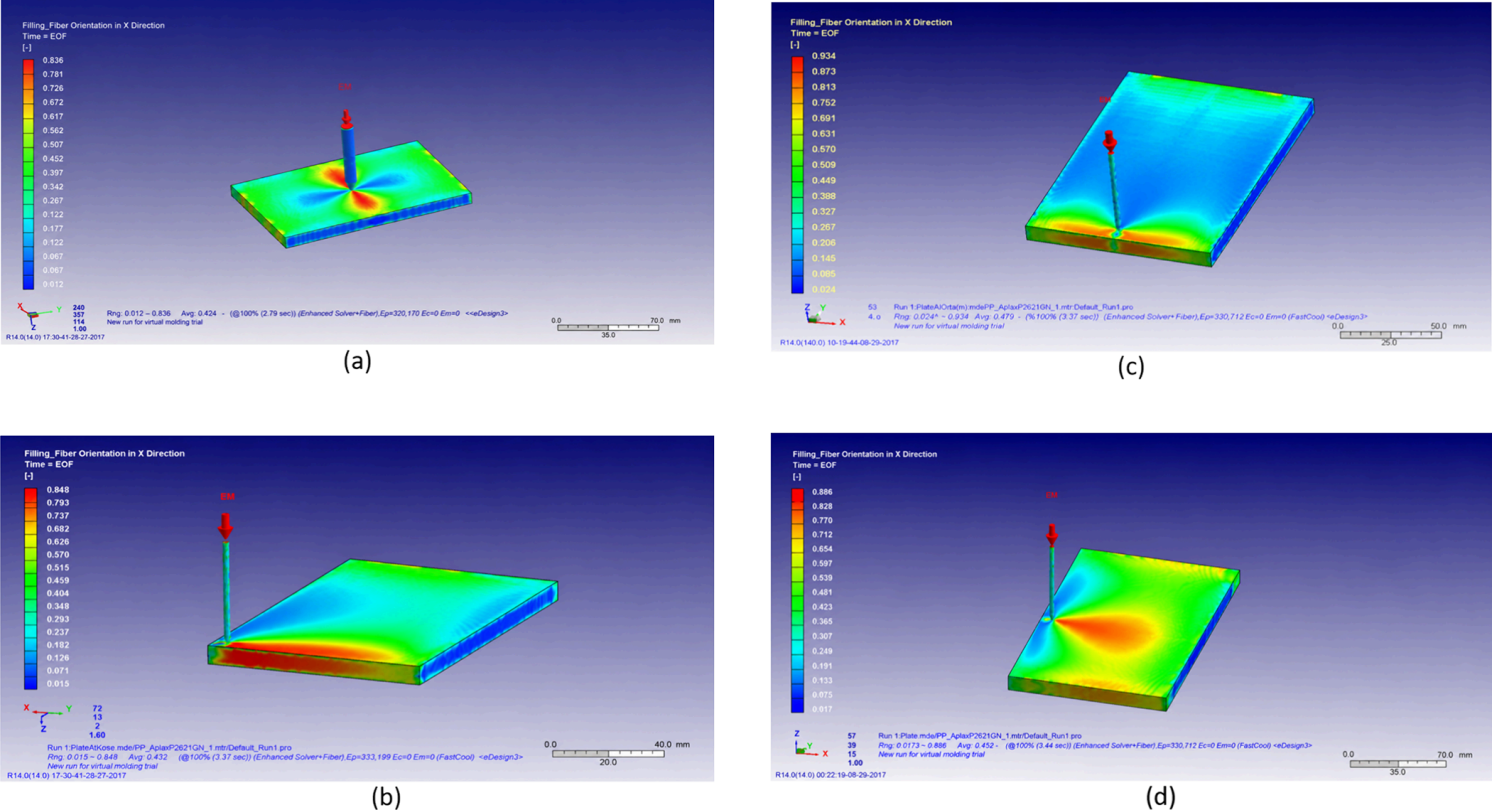
Fiber orientation distributions in the X direction for POK–20CF composite under middle (a), corner (b), short side (c), and long side (d) feeding locations.

**5:**
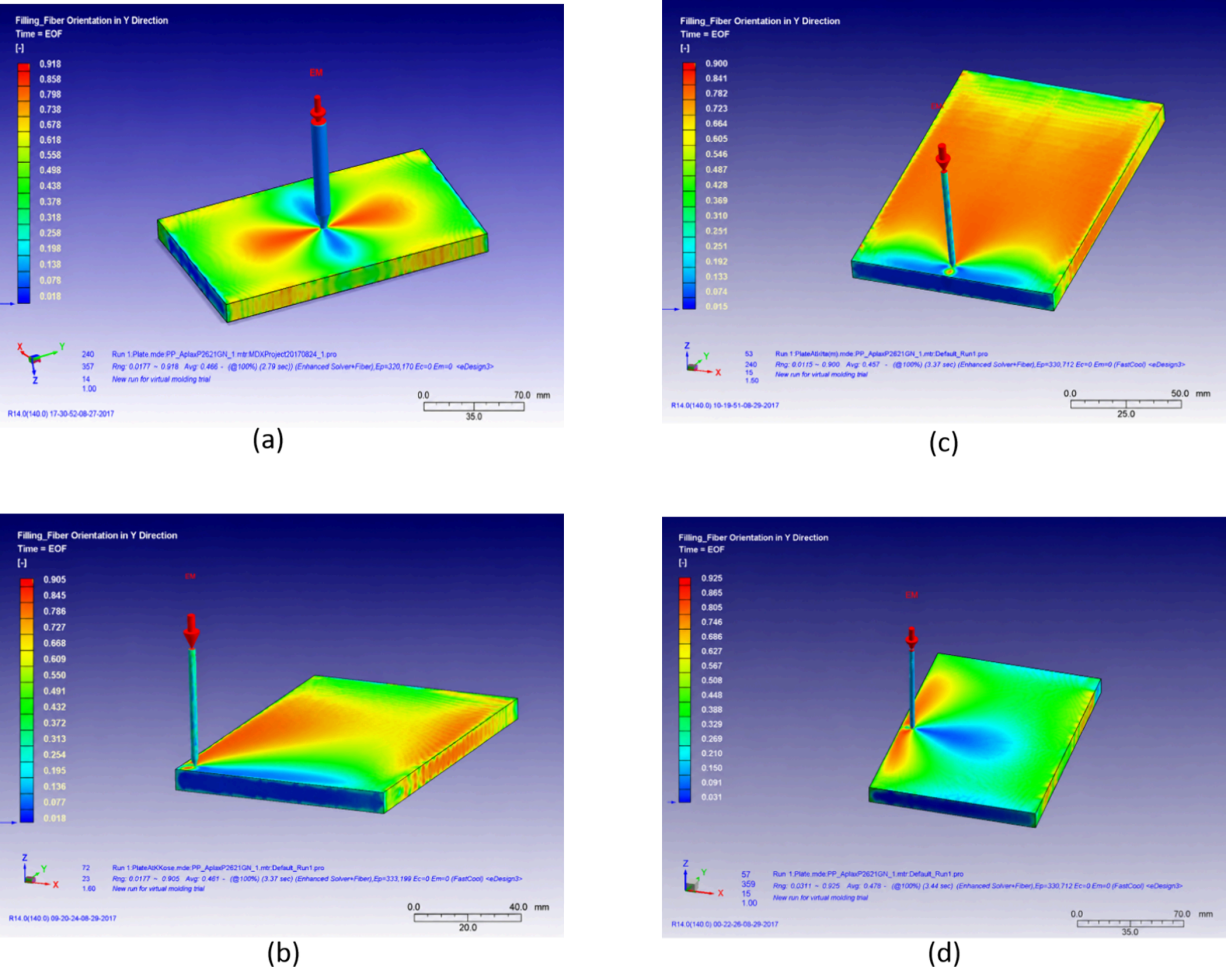
Fiber orientation distributions in the Y direction for POK–20CF composite under middle (a), corner (b), short side (c), and long side (d) feeding locations.

**Figure 6:**
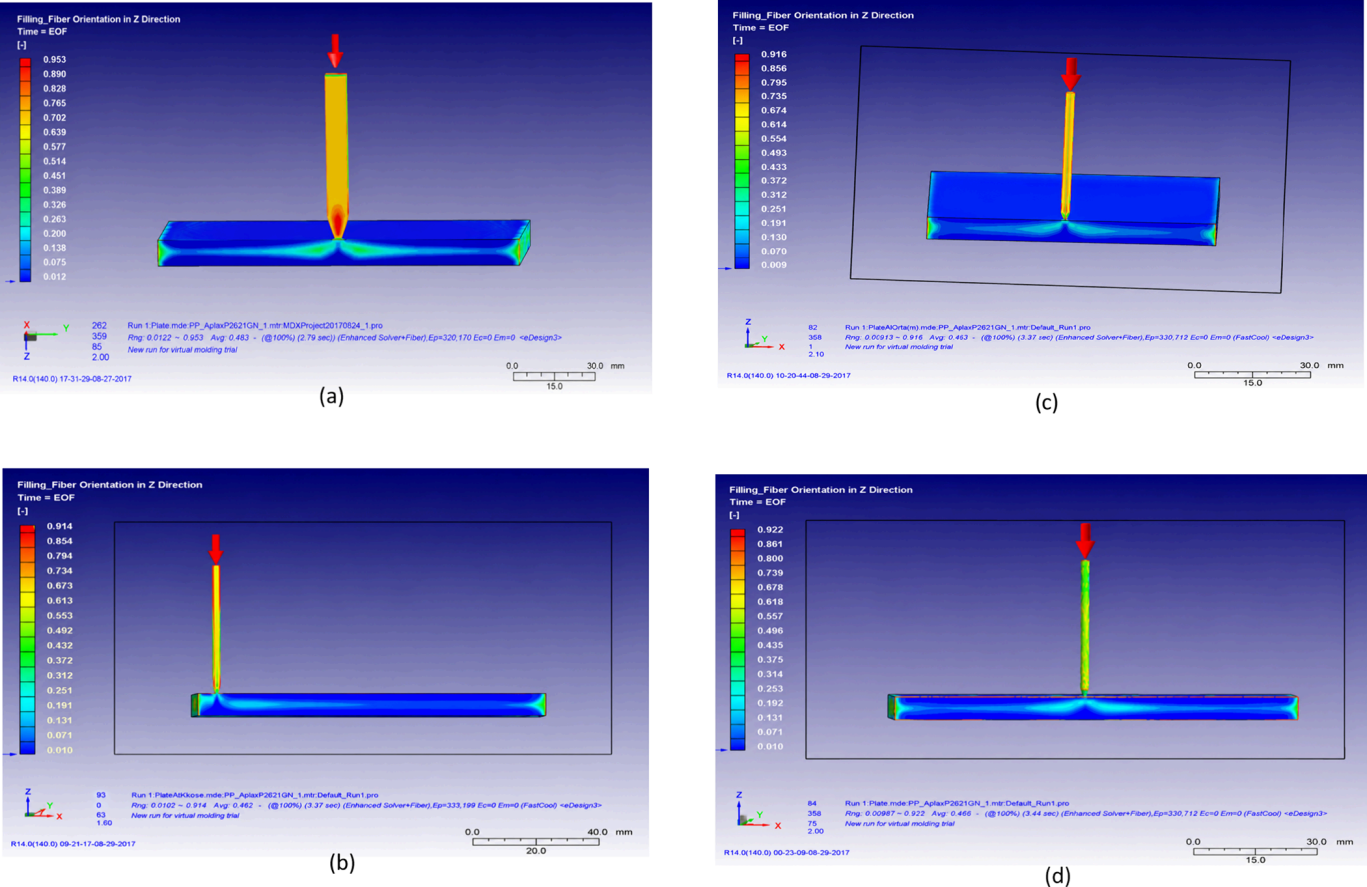
Fiber orientation distributions in the *Z* direction for POK–20CF composite under middle (a), corner (b), short side (c), and long side (d) feeding locations.

The orientation angles obtained from these simulations were used as inputs for the mathematical model. The model was implemented and solved using MATLAB, and the resulting thermal conductivity values were compared with experimental measurements. A summary of both the numerical and experimental results is presented in Tables 2 and[Table tbl3] for in-plane thermal conductivity and Tables 4 and 5 for through-plane thermal conductivity.

**Table 2:** In-Plane Thermal Conductivity Values (W/m·K) of POK-Based Composite Materials Obtained Experimentally and Numerically

		numerical values			
**samples**	experimental values (flow 3)	flow 1	flow 2	flow 3	flow 4
**POK**	0.21 ± 0.01	0.21	0.21	0.21	0.21
**POK-10SG**	2.17 ± 0.20	2.20	2.19	2.20	2.21
**POK-20SG**	3.97 ± 0.32	3.95	3.96	3.97	3.98
**POK-30SG**	7.02 ± 0.65	7.11	7.09	7.05	7.12
**POK-40SG**	9.16 ± 0.95	9.15	9.14	9.16	9.17
**POK-10CF**	1.98 ± 0.02	2.11	1.89	2.34	1.05
**POK-20CF**	3.54 ± 0.04	3.47	2.43	3.61	1.52
**POK-30CF**	6.85 ± 0.08	6.57	4.78	6.86	3.24
**POK-40CF**	8.54 ± 0.07	8.23	6.91	8.51	5.34
**POK-1CNT**	0.77 ± 0.06	0.76	0.77	0.76	0.75
**POK-2CNT**	1.05 ± 0.12	1.04	0.99	1.03	1.04
**POK-3CNT**	1.94 ± 0.02	1.92	1.93	1.94	1.94
**POK-1BN**	0.45 ± 0.03	0.45	0.44	0.45	0.44
**POK-2BN**	0.98 ± 0.07	0.97	0.98	0.97	0.98
**POK-3BN**	1.12 ± 0.10	1.11	1.11	1.12	1.12
**POK-1.5BN-1.5CNT**	1.57 ± 0.12	1.57	1.56	1.57	1.57

**Table 3:** Examples of Different Combinations Generated by the Model

	numerical values			
**samples**	flow 1	flow 2	flow 3	flow 4
**POK-20CF-10SG**	4.65	5.83	3.72	5.12
**POK-20CF-20SG**	5.63	7.81	4.02	6.73
**POK-20CF-20SG-1CNT**	8.45	10.23	6.71	9.76
**POK-20CF-20SG-3CNT**	10.32	13.87	7.01	12.82

**Table 4:** Comparison of Experimentally Measured and Numerically Predicted Through-Plane Thermal Conductivity Values (W/m·K) of POK-Based Composites

		numerical values			
**samples**	experimental values (flow 3)	flow 1	flow 2	flow 3	flow4
**POK**	0.21 ± 0.01	0.21	0.21	0.21	0.21
**POK-10SG**	0.35 ± 0.02	0.35	0.35	0.35	0.35
**POK-20SG**	0.48 ± 0.04	0.48	0.48	0.48	0.48
**POK-30SG**	0.71 ± 0.06	0.71	0.71	0.71	0.71
**POK-40SG**	0.92 ± 0.07	0.92	0.92	0.92	0.92
**POK-10CF**	0.41 ± 0.03	0.41	0.41	0.41	0.41
**POK-20CF**	0.54 ± 0.05	0.54	0.54	0.54	0.54
**POK-30CF**	0.87 ± 0.07	0.87	0.87	0.87	0.87
**POK-40CF**	1.12 ± 0.11	1.12	1.12	1.12	1.12
**POK-1CNT**	0.37 ± 0.03	0.37	0.37	0.37	0.37
**POK-2CNT**	0.51 ± 0.04	0.51	0.51	0.51	0.51
**POK-3CNT**	0.72 ± 0.07	0.72	0.72	0.72	0.72
**POK-1BN**	0.34 ± 0.02	0.34	0.34	0.34	0.34
**POK-2BN**	0.41 ± 0.03	0.41	0.41	0.41	0.41
**POK-3BN**	0.49 ± 0.04	0.49	0.49	0.49	0.49
**POK-1.5BN-1.5CNT**	0.5 ± 0.04	0.5	0.5	0.5	0.5

**Table 5:** Examples of Different Filler Combinations Predicted by the Model

	numerical values			
**samples**	flow 1	flow 2	flow 3	flow 4
**POK-20CF-10SG**	0.68	0.68	0.68	0.68
**POK-20CF-20SG**	0.82	0.82	0.82	0.82
**POK-20CF-20SG-1CNT**	0.94	0.94	0.94	0.94
**POK-20CF-20SG-3CNT**	1.12	1.12	1.12	1.12

To improve the quantitative comparison between experimental measurements and numerical predictions, the results are presented in graphical form in addition to tabulated data. Figures 7 and 8 show plots comparing experimentally measured and numerically predicted in-plane and through-plane thermal conductivity values, respectively.

**Figure 7:**
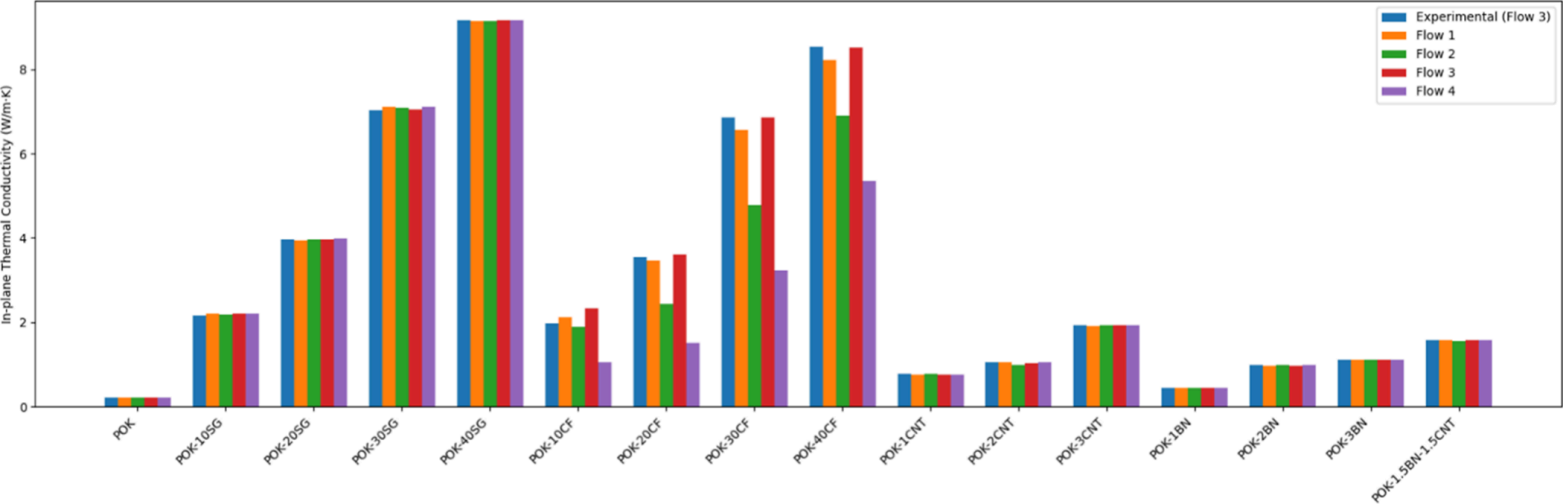
Comparison of experimentally measured (flow 3) and numerically predicted (flows 1–4) in-plane thermal conductivity values of POK-based composites with different filler systems.

**Figure 8:**
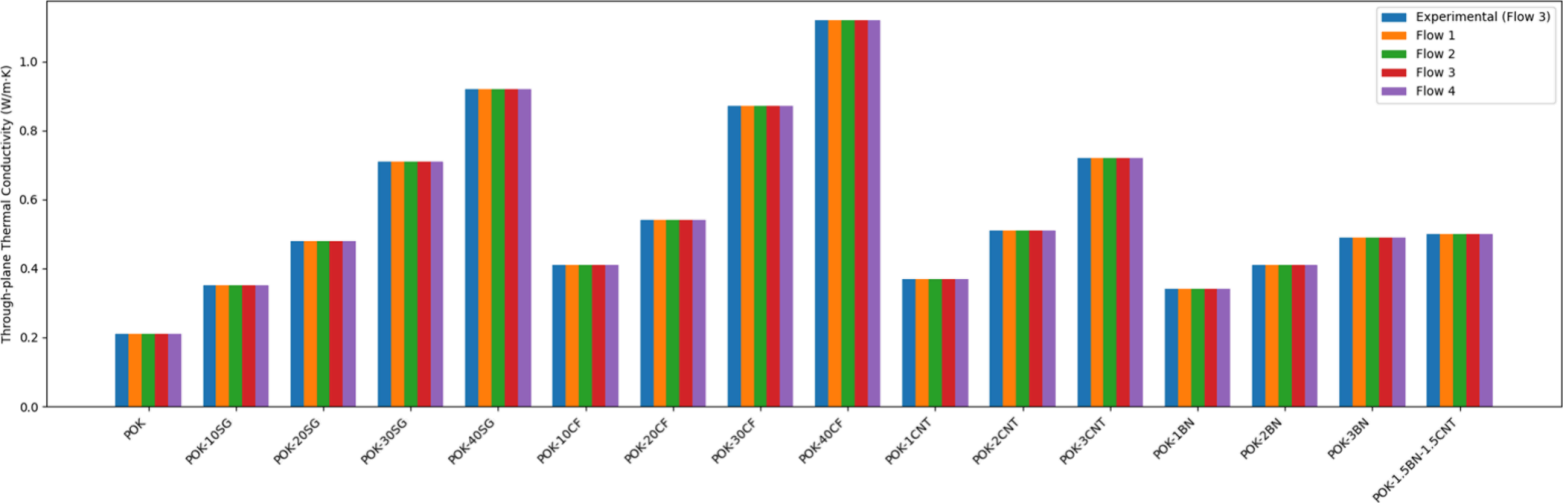
Comparison of experimentally measured (flow 3) and numerically predicted (flows 1–4) through-plane thermal conductivity values of POK-based composites with different filler systems.

The graphical comparison shows strong agreement between model predictions and experimental results across all filler systems, with deviations within ± 10% for fiber-filled composites and ± 5% for particle-filled systems. The largest discrepancies were observed for carbon fiber–reinforced composites under Flow Directions 1 and 2, where pronounced fiber misalignment reduces effective in-plane conductivity..

Based on the results, the injection gate location used for the experimentally produced specimens corresponds to Flow Direction 3. As expected, the injection direction plays a critical role in determining the in-plane thermal conductivity of carbon fiber–reinforced composites, primarily because fibers are strongly aligned along the melt flow path. In contrast, composites containing particulate fillers exhibit significantly less dependence on injection direction, as particle alignment is far less sensitive to flow-induced orientation effects. Unlike in-plane conductivity, through-plane thermal conductivity is insensitive to injection flow direction, yielding identical numerical predictions across all gate configurations. These results confirm that fiber orientation, governed primarily by gate location and melt flow direction, is the dominant factor influencing anisotropic thermal conduction in CF-reinforced POK composites, whereas particulate fillers such as SG, BN, and CNT exhibit significantly lower sensitivity to processing-induced orientation effects.

## 4 Conclusions

This study systematically investigated the thermal conduction behavior of POK-based composites reinforced with SG, CF, CNT, BN, and hybrid BN–CNT fillers. A comprehensive three-dimensional thermal conductivity model was developed that integrates filler anisotropy, morphology, and orientation with multifiller synergy parameters. Experimental results and model predictions yielded several key conclusions:

1. Significant Thermal Conductivity Enhancement: The incorporation of thermally conductive fillers markedly improved both in-plane and through-plane conductivity. SG yielded the highest in-plane values, whereas CF enabled more balanced heat conduction pathways.
2. Filler Morphology Dictates Anisotropy: Plate-like fillers, such as SG and BN, exhibited strong alignment during melt flow, resulting in highly anisotropic conduction. In contrast, fibrous CF facilitated the formation of interconnected networks, thereby enhancing conductivity in both directions.
3. Hybrid Fillers Demonstrate Synergistic Behavior: In-plane thermal conductivity increased from 0.21 W/m·K for neat POK to 9.16 W/m·K for the POK–40SG composite, while the POK–40CF composite achieved 8.54 W/m·K in-plane and 1.12 W/m·K through-plane conductivity. In addition, the BN–CNT hybrid system demonstrated enhanced thermal conductivity compared with the individual filler systems at equivalent total loading, indicating synergistic network formation..
4. **I**njection Molding Parameters Are Critical for Fiber-Filled Composites: The gate location significantly influenced fiber orientation and, consequently, the thermal conductivity in CF-reinforced samples. In contrast, particle-filled composites were less sensitive to flow direction.
5. Validation of the Proposed 3D Model: Numerical predictions closely matched experimental data for both single- and multifiller systems, confirming the model’s reliability and suitability for predicting thermal conductivity in anisotropic, hybrid composites. The proposed 3D model predicted experimental thermal conductivity values with deviations generally below ± 10% for CF-containing systems and below ± 5% for particle-filled composites.
6. Implications for Advanced Thermal Management: The findings offer valuable design guidelines for developing lightweight, high-performance composite materials for thermal management in electronics, automotive systems, and aerospace structures.

## Future Work

Future research should focus on integrating interfacial thermal resistance into the model, exploring percolation thresholds in multifiller networks, studying long-term thermal stability and mechanical–thermal coupling, and extending the model to predict thermal conductivity under dynamic thermal loading.
